# Artificial intelligence for classifying uncertain images by humans in determining choroidal vascular running pattern and comparisons with automated classification between artificial intelligence

**DOI:** 10.1371/journal.pone.0251553

**Published:** 2021-05-14

**Authors:** Shozo Sonoda, Hideki Shiihara, Hiroto Terasaki, Naoko Kakiuchi, Ryoh Funatsu, Masatoshi Tomita, Yuki Shinohara, Eisuke Uchino, Takuma Udagawa, Guangzhou An, Masahiro Akiba, Hideo Yokota, Taiji Sakamoto

**Affiliations:** 1 Department of Ophthalmology, Kagoshima University Graduate School of Medical and Dental Sciences, Kagoshima, Japan; 2 Sonoda Eye Clinic, Kagoshima, Japan; 3 R&D Division, Topcon Corporation, Tokyo, Japan; 4 Image Processing Research Team, RIKEN Center for Advanced Photonics, RIKEN, Wako, Japan; University of Florida, UNITED STATES

## Abstract

**Purpose:**

Abnormalities of the running pattern of choroidal vessel have been reported in eyes with pachychoroid diseases. However, it is difficult for clinicians to judge the running pattern with high reproducibility. Thus, the purpose of this study was to compare the degree of concordance of the running pattern of the choroidal vessels between that determined by artificial intelligence (AI) to that determined by experienced clinicians.

**Methods:**

The running pattern of the choroidal vessels in en face images of Haller’s layer of 413 normal and pachychoroid diseased eyes was classified as symmetrical or asymmetrical by human raters and by three supervised machine learning models; the support vector machine (SVM), Xception, and random forest models. The data from the human raters were used as the supervised data. The accuracy rates of the human raters and the certainty of AI’s answers were compared using confidence scores (CSs).

**Results:**

The choroidal vascular running pattern could be determined by each AI model with an area under the curve better than 0.94. The random forest method was able to discriminate with the highest accuracy among the three AIs. In the CS analyses, the percentage of certainty was highest (66.4%) and that of uncertainty was lowest (6.1%) in the agreement group. On the other hand, the rate of uncertainty was highest (27.3%) in the disagreement group.

**Conclusion:**

AI algorithm can automatically classify with ambiguous criteria the presence or absence of a symmetrical blood vessel running pattern of the choroid. The classification was as good as that of supervised humans in accuracy and reproducibility.

## Introduction

Automatic classification of normal and diseased conditions by artificial intelligence (AI) is becoming more widely used [1–3]. This is especially true in the field of ophthalmology where a diagnostic device with AI algorithm was first approved by the US FDA in 2018 [4–7]. It was expected that AI would become more helpful and popular in classifying ocular diseases especially those with ambiguous signs and symptoms [8].

An example of the difficulty of subjective classification can be found in the analyses of the choroid. The choroid is an important tissue that is involved in the pathogenesis of many vitreoretinal diseases, such as age-related macular degeneration and central retinal chorioretinopathy [9]. However, it is not easy to evaluate the vascular pattern quantitatively because the choroid is composed mainly of vascular tissue without an ordered architecture as the retina.

The pachychoroid spectrum disorders are new disease entities that are characterized by a thickened choroid [10, 11]. However, the pachychoridal disorders have other morphological changes of the choroid. For example, Hayreh and other investigators reported that the watershed zone of the choroid in normal eyes is located mainly along a line connecting the macula to the optic disc. Then, the blood vessels of Haller’s layer of the choroid in the posterior pole have a symmetrical structure with the two regions separated by this horizontal line [12–14]. Other researchers and our laboratory have focused on the vascular structure of the watershed zone of Haller’s layer in the en-face images obtained by optical coherence tomography angiography (OCTA) in different retinocortical disorders [15]. Kishi et al. reported that the vessels in Haller’s layer in eyes with central serous chorioretinoapthy (CSC) were more asymmetrical than that of normal eyes [16]. Savastano et al. evaluated the vascular pattern of Haller’ layer and found that the percentage of eyes with a symmetrical vessel pattern was higher in normal eyes, and the percentage of eyes with an asymmetrical pattern of flow was higher in CSC eyes [17]. These are important findings that can contributed to the understanding of the pathogenesis of CSC. However, the analysis used to determine the symmetry was subjective and thus the reproducibility was not good.

To overcome this limitation, we have adopted AI methods to do the analyses. If AI can attain reproducible classification of ambiguous patterns as determined by subjective criteria, the classification would become more reliable.

Therefore, the purpose of this study was to determine whether AI methods can be used to detect and quantify the symmetry of the running pattern of the choroidal vessels. To accomplish this, we used three different AI models and compared their results to that of humans.

## Methods

This study was approved by the Ethics Committee of Kagoshima University Hospital (Kagoshima, Japan) and registered with the University Hospital Medical Network (UMIN)-clinical trials registry (CTR). The registration title is “UMIN000031747, Research on retinal/choroidal structure analysis by novel image analysis technique and machine learning.” on March 2018. A detailed protocol is available at, https://upload.umin.ac.jp/cgi-open-bin/ctr/ctr_view.cgi?recptno=R000036250. A written informed consent was obtained from all of the subjects after an explanation of the procedures to be used and possible complications. All of the procedures conformed to the tenets of the Declaration of Helsinki. The optical coherence tomographic (OCT) data were recorded with a protocol approved by the Institutional Review Board (IRB) (W2017-084).

This was a retrospective study of total 451 subjects of normal eyes and eyes with pachychoroid spectrum disease who belonged to patients at the Kagoshima University Hospital. The patients were examined from March 2017 to December 2018, and the data from only one eye of each individual were used for the statistical analyses.

The classification of pachychoroid disease was based on the criteria of earlier reports [9, 11]. Eyes with other ocular diseases such as glaucoma, diabetic retinopathy, or with prior intraocular surgery or injections were excluded. Also excluded were eyes whose OCT image quality was ≤80, with myopia of ≤ -6.0 diopters (D) because the possible presence of altered choroidal structure, with a fovea away from the center of the image because of poor fixation, and with inaccurate segmentation of Bruch’s membrane.

All of the eyes had a comprehensive ocular examination including slit-lamp examinations of the anterior segment and ophthalmoscopic examinations of the fundus. The axial length was measured with the AL-2000 ultrasound instrument (Tomey, Tokyo, Japan). The best-corrected visual acuity (BCVA) was measured after determining the refractive error with an Auto Kerato-Refractometer (RM8900, Topcon). Only the data from the right eyes were statistically analyzed.

### Image protocol and selection of en face image of Haller’s layer

A swept-source OCT instrument (DRI OCT Triton, Topcon) with a light source whose center wavelength was 1050 nm was used to record the images. The scanning speed was 100,000 A-scans/s with an 8 μm axial and a 20 μm transverse resolution. The 3D-Macula scan mode with scans of 7×7 mm centered on the macula was used. Each OCT image was the average of 4 scans. The EnView software (Topcon) was used to flatten the B-scan images relative to Bruch’s membrane, and averaging was performed on the images before and after the flattening. These procedures led to the creation of en face images with a thickness of 2.6 μm for each slab, and these images were used to create 512 × 512 pixels bit map images. The slab selected for the analyses was the top 25% slab of the whole Haller’s layer which was automatically selected using the AI-software as we described in detail in an earlier publication [18]. Briefly, OCT en face images of the choroid were obtained every 2.6 μm from the retinal pigment epithelium (RPE) to the chorioscleral border. The images at the beginning of the choriocapillaris, the start of Sattler’s layer, and the start of Haller’s layer were identified. The image numbers from the RPE border were taken as the teacher’s data. The values of 41 feature qualities of each image were extracted. A machine learning model was created from the value of each feature of the training data. The software developed to accomplish this can automatically detect the boundaries of the choriocapillaris, Sattler’s layer, and Haller’s layer in an objective way. After determining each boundary with this software, an en face image from the superficial 25% layer of Haller’s layer was selected for the analyses.

### Supervised data on symmetry of running pattern of blood vessels

**Fig 1:**
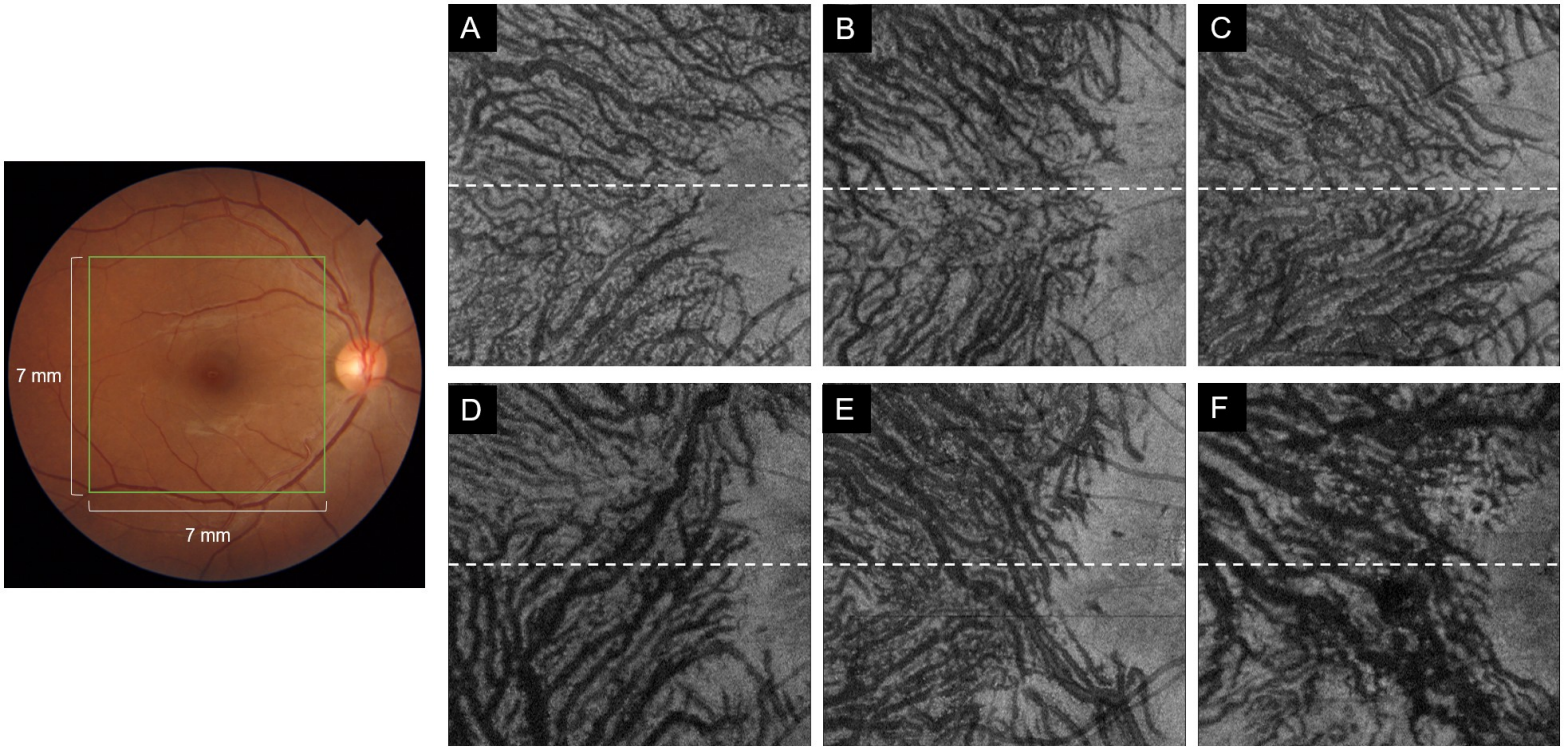
Representative en face images of the top 25% slab in Haller’s layer of the right eye. In the image, the macula and optic disc are located on a horizontal line that passes through the center of the images. Upper row (**A**, **B**, **C**) are representative cases of a symmetrical pattern. The vessel running pattern is symmetrical to the macula and optic disc as the center. Lower row (**D**, **E**, **F**) are representative cases of asymmetric pattern. None of the upper or lower blood vessel extends significantly beyond the centerline to interrupt the symmetrical structure.

The supervised data obtained from the en face images of Haller’s layer were classified as symmetrical or asymmetrical (Fig 1). All of the images were annotated by two retinal specialists and if a disagreement was obtained, it was removed from the study.

### Establishment of AI-powered model

**Fig 2:**
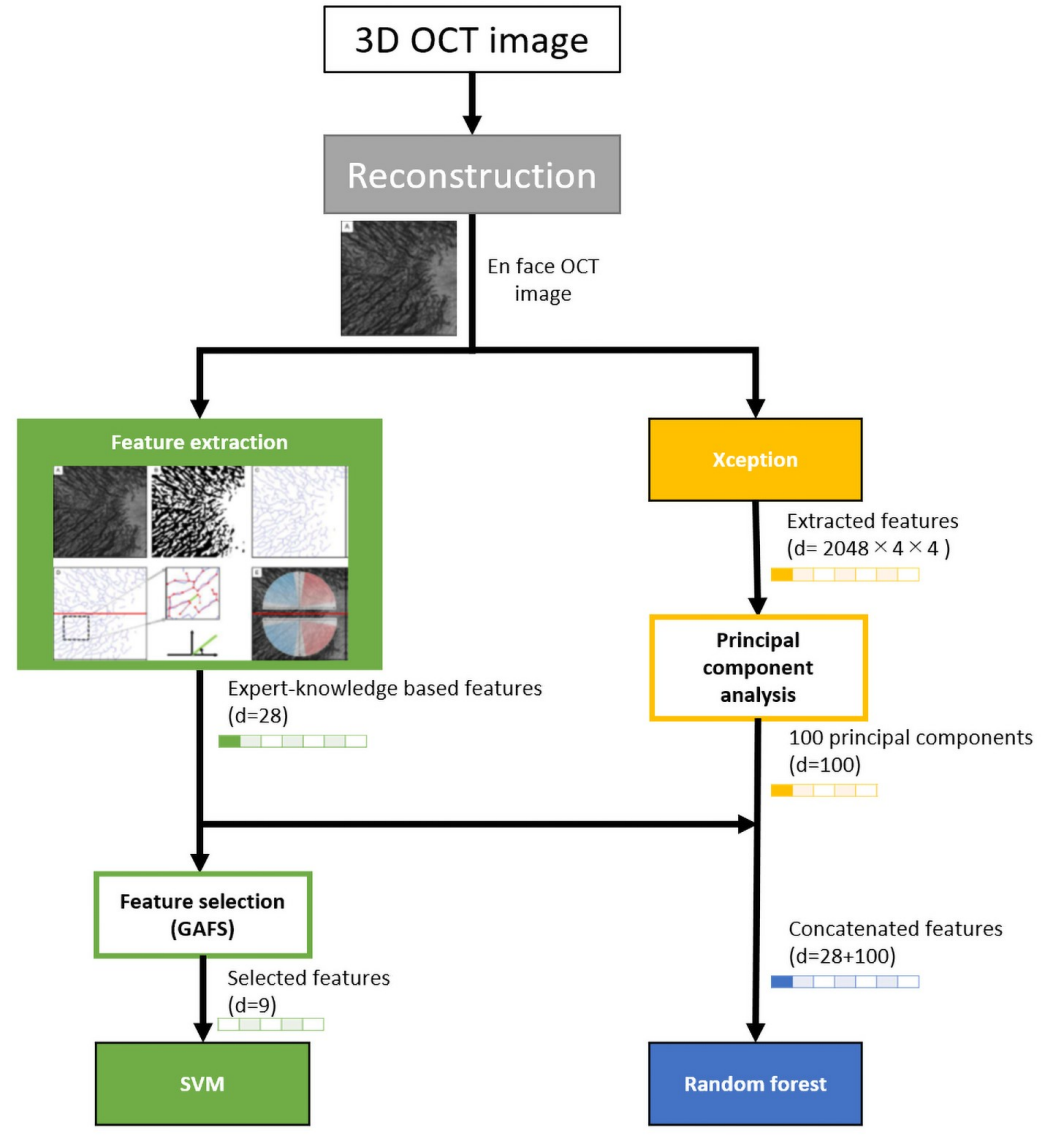
The flow to create the SVM, Xception, and Random Forest supervised learning models. In the SVM model, the feature quantities were selected using our original en face image quantification and used for training. In the Xception model, only en face images were inputted, and in the Random Forest model, the image features used in the other two models were used for learning.

Three AI-powered models were built. The first was a support vector machine (SVM) model based on the numerical data from the en face image of the top 25% slab of Haller’s layer as described in detail [15, 18]. The second was the Xception model whose input was the same en face images that were used for SVM. The third was the random forest model whose input were the data used in SVM and that extracted from the trained Xception model. The flow to create the three models are shown in Fig 2. To validate each model, the data were randomly divided by 10-folds and a cross validation of the 10-fold split was performed. In addition, we compared the three models by applying Wilcoxon signed-rank test of the area under the receiver operating characteristic curve (AUC) and determined the accuracy of this. All three models were standardized supervised learning models.

### SVM model and numerical data extraction from choroidal en face images

In the analyses of the en face images of Haller’s layer, the blood vessels were displayed as black, and the characteristics of the structure of the choroidal blood vessel were quantified using our software to obtain the features of the images [15]. Briefly, the selected en face image was binarized and de-noised to separate vasculature from the other structures. Then, a skeletonizing process was performed to detect the center of the blood vessels. The blood vessel area, length, and diameter were calculated by this process. In a normal eye, there is a watershed line connecting the macula and the optic disc [12], and it divides the upper and lower posterior pole area hemodynamically. From this, the quantitative numerical values related to the blood vessel structure were calculated as displayed in Table 1. Then, the upper and lower ratios and differences were calculated and used as the image features.

**Table 1:** Quantified parameters based on en-face image analyzer.

No.	Features	No.	Features
1	vessel area, upper side (mm^2^)	15	Ratio of natural to unnatural oblique vessel, upper side
2	vessel length, upper side (mm)	16	Ratio of natural to unnatural oblique vessel, lower side
3	mean vessel diameter, upper side (mm)	17	Ratio of No.15 feature to No.16 feature
4	vessel area, lower side (mm^2^)	18	Difference of No.15 feature between No.16 feature
5	Vessel length, lower side (mm)	19	Upper to lower ratio of natural oblique vessel
6	mean vessel diameter, lower side (mm)	20	Upper to lower difference of natural oblique vessel
7	Upper to lower ratio of mean vessel diameter	21	Upper to lower difference of mean vessel diameter, ladder pattern image (mm)
8	Upper to lower difference of mean vessel diameter	22	Upper to lower difference of total vessel length, ladder pattern image (mm)
9	ratio of vessels flowing from upper temporal side to lower nasal side	23	Upper to lower ratio of mean vessel diameter, ladder pattern image (mm)
10	ratio of vessels flowing from upper nasal side to lower temporal side	24	Upper to lower ratio of total vessel length, ladder pattern image (mm)
11	ratio of natural oblique vessel, upper side (%)	25	Temporal to nasal difference of mean vessel diameter, ladder pattern image (mm)
12	ratio of unnatural oblique vessel, upper side (%)	26	Temporal to nasal difference of total vessel length, ladder pattern image (mm)
13	ratio of natural oblique vessel, lower side (%)	27	Temporal to nasal ratio of mean vessel diameter, ladder pattern image (mm)
14	ratio of unnatural oblique vessel, lower side (%)	28	Temporal to nasal ratio of total vessel length, ladder pattern image (mm)

Next, the blood vessel bifurcations were automatically detected in the skeletonized images, and a blood vessel was defined as a set of line segments connecting the bifurcation points. Each line segment was noted to project in one of eight directions; upper and lower, nasal, temporal, nasal upper, and lower side, temporal upper, and lower sides. The vector of the line segments was used to quantify the blood vessel running direction.

**Fig 3:**
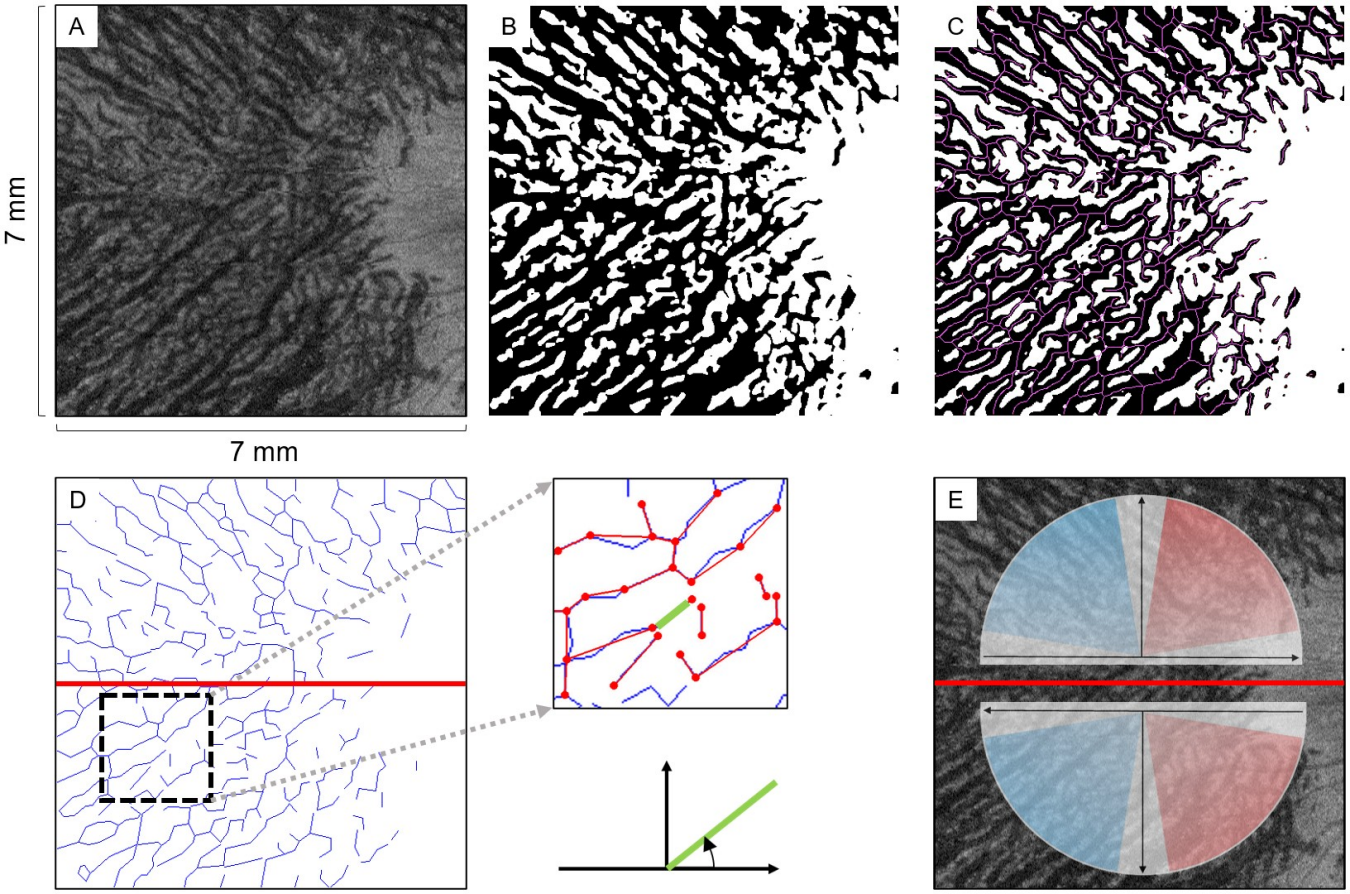
Image processing procedures. The en face image of the superficial 25% slab of Haller’s layer of the right eye that was selected automatically (**A**). Display of the vessel area and other components were separated by the binarization method (**B**). The vessels were thinned by the Zhang-Suen thinning algorithm which made it possible to obtain numerical data on the blood vessels length and diameter (**C**). To quantify the vessel running pattern, the en face image was divided evenly into the upper and the lower regions relative to the position of the watershed zone of a normal eye. The thinned blood vessel is a group of line segments divided by the blood vessel branches, and the blood vessel running was quantified by measuring the angle of each line segment (**D**). The upper region from the macula to the upper temporal side (lower right to upper left) is defined as the natural oblique vessel flow, and the blood vessels at an angle of 95° to 175° are classified as “upper natural oblique vessels”. In the lower region, from the macula to lower temporal side (upper left to lower right) is the natural blood flow, and blood vessels at an angle of 5° to 85° are classified as “lower natural oblique vessels” (**E**).

The blood in the vessels of Haller’s layer flowed from the macular region to the upper temporal vortex vein, and it also flowed to the lower temporal vortex vein in the lower region if the choroidal vessels were vertically symmetrical [14]. Thus, the direction from the macula to the upper temporal vortex vein, i.e., from the lower right to the upper left for the right eye in the upper region, and the direction from the macula to the lower temporal vortex vein, i.e., from the upper right to the lower left for the right eye in the lower region were classified as "natural oblique vessels". The blood vessels running in the opposite direction were classified as "unnatural oblique vessels" (Fig 3, S1 Fig) [15].

**Fig 4:**
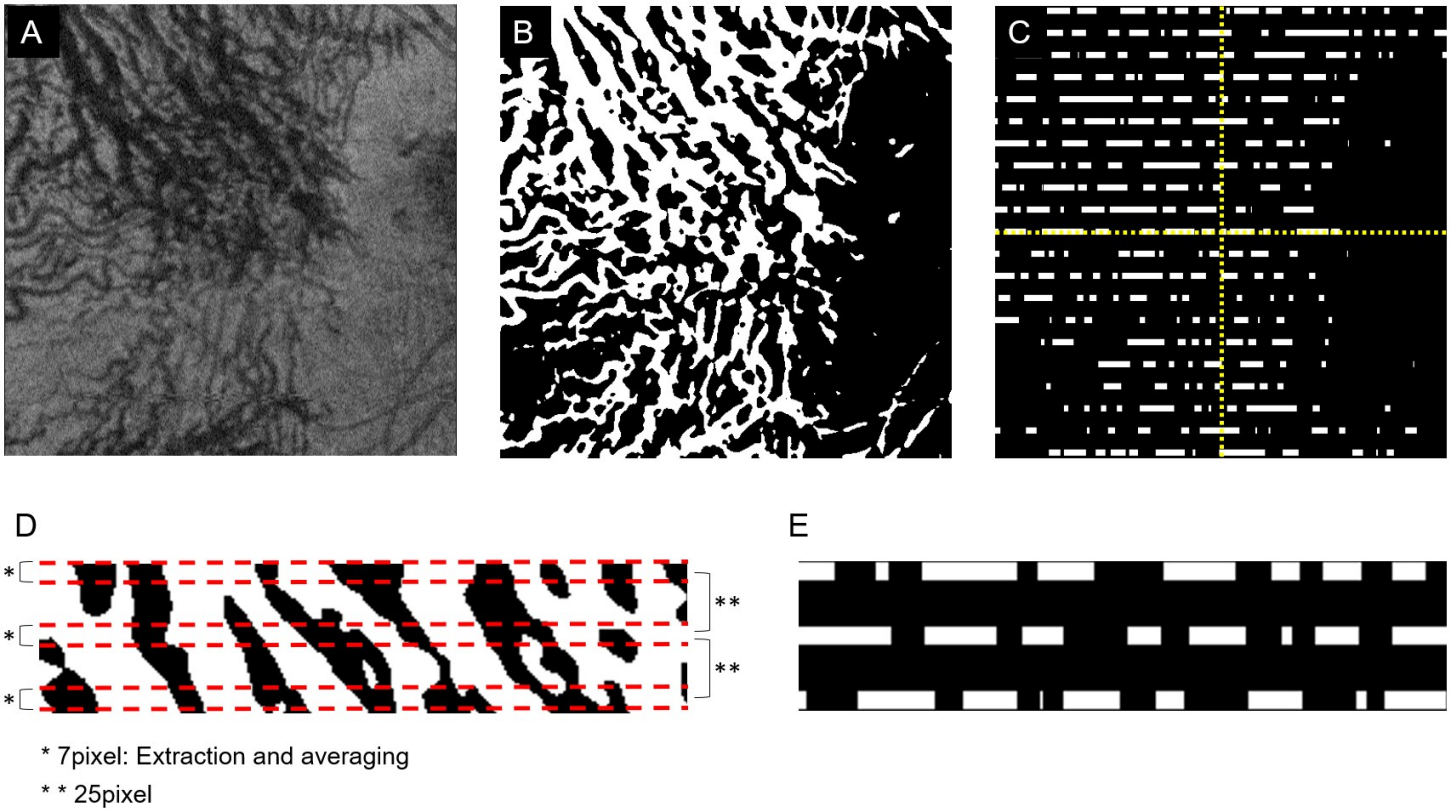
Representitive image of converted blood vessel runnning to ladder pattern image. **A**: En face image of the top 25% slab of Haller’s layer. **B**: Image was binarized by the discriminant analysis method, and the choroidal vessels are drawn in white. **C**: The binary image is converted to ladder pattern image. **D**: and **E**: A line was drawn in the horizontal direction every 25 pixels, and the blood vessel components within a range of 7 pixels width were extracted around the line segment and converted to a white line as a ladder pattern image. The total length and the mean diameter of the blood vessel were calculated based on it, and the vertical difference and the difference between the nasal side and the temporal side were calculated and used as image features. With these methods, 28 image features of the choroidal en face images were determined and used for the AI analyses.

Next, a ladder pattern image was created to determine if trends such as the variations in the size of blood vessels were present in the binarized image. A line was drawn for every 25 pixels of the binarized image, and then a 7-pixel strip was created and only this strip was extracted (Fig 4). If the majority of the vertical line within the 7-pixel strip was white, it was labeled white, and if the majority was black, it was labeled black. The result is a ladder pattern image that captures the trend of the image (Fig 4).

The 28 quantified ocular parameters for each eye after rudimentary judgement of various ocular parameters are shown in Table 1. We explored the SVM, which is a common used supervised machine-learning classifier [19, 20], with radial basis function (RBF) kernels to classify the en face images with these 28 features. A genetic-algorithm-based feature selection (GAFS) using randomness that mimics natural evolution [21] was used to find the most valid features of the SVM. The parameters used for the GAFS were a population size of 20, crossover probability of 0.7, mutation probability of 0.2, tournament size of 2, and number of generations of 1000 using early stopping.

### Xception with original en-face image

Xception is a well-known architecture of convolutional neural networks (CNN) [22]. The input data of Xception is only the en face images, and the numerical data were not used as they were with SVM. Note that we used the en-face images resized from 512 × 512 pixels to 128 × 128 pixels. This model is independent of the expert assessments.

Transfer learning from a Xception model pretrained on ImageNet dataset was applied. We also applied some data augmentation methods, e.g., horizontal flip, vertical flip, and ±15 degree rotation.

### Random Forest analyses with data used in SVM and data extracted from Xception

The CNN model consisted of a feature extraction unit and a classifier unit [23]. Xception was no exception. We extracted 2048 × 4 × 4 feature quantities from the last layer of the feature extraction unit of a trained Xception. We also reduced the dimension of the extracted features to 100 by principal component analysis (PCA). We trained the random forest model with 100000 trees using both the 28 feature quantities designed by the doctors and 100 new features created from the Xception model.

All three models including the SVM, Xception, and Random Forest models were implemented in Python (ver.3.6.5) on Ubuntu 16.04 OS, and trained with one Nvidia Quadro GV100 GPU, Intel Xeon Gold 6130 CPU, and 96 GB memory. To show the effectiveness of our proposed three models, a 10-fold cross validation method was applied on the entire dataset (n = 413). As a result, for each crossover experiment, approximately 372 images were used as the training data which were applied with the data augmentation method for each epoch during the entire training period of 2,000 epochs, while 41 images were used as test data. The SVM and Random Forest models were implemented with the scikit-learn program (ver.0.20.3), while the deep learning model (parameters of Xception are totally 37,772,841 after modification increased from the original number 22,910,480) is implemented in Python (ver.3.6.5) with TensorFlow (ver.1.13.1) as the backend in the Keras (ver.2.2.4) The optimizer is the Stochastic Gradient Descent (SGD) method, with a batch size of 32, a learning rate of 0.0001, and a momentum of 0.9. The loss function used for the Xception model is binary cross-entropy. The training was completed after 2,000 epochs for each fold in approximately 1 hour. The model used at test time is an average of all the samples in test dataset 81 ms/image.

### Comparisons of judgment ability of humans and AI models on vascular running pattern of Haller’s layer

The judgment by AI was designated by the confidence score (CS) which quantitatively indicated the confidence of the judgments [24, 25]. With this score, we compared how humans judged the events that AIs were not sure about. This time, the random forest had the highest classified ability, so we tested it. The CS value was between 0 and 1, and the closer the CS was to 0, the more confident it was to be asymmetrical, and the closer the CS was to 1, the more confident it was to be symmetrical. It is important to note that 0.9 is confident that the symmetry is present, and 0.1 is confident that the symmetry is not present. Thus, the closer the index was to 0.5, the less confident was the decision. Therefore, the absolute value of the difference from 0.5 was assumed to be the "index of strength of self-confidence". In the index of strength of self-confidence using CS, 0 to 0.166 of CS is defined as “unconfident”, 0.167 to 0.332 is “moderately confident”, and 0.333 to 0.5 is “confident”.

To determine whether there is confidence in the classification by humans, the following grouping was performed. The results obtained by an agreement of the classification by the two graders were used as supervised data. Three independent retina specialists other than the supervised data creators made a classification of each eye. Therefore, the group that matched the classification of the supervised data with all three was categorized as the “agreement group”. An agreement between the two evaluators and the supervised data was defined as the “partial agreement group”. Only one evaluator that matched the supervised data was set as the “partial disagreement group”. The “disagreement group” was those in which all three evaluators disagreed with the supervised data.

The confidence of humans was divided into four categories: agreement, partial agreement, partial disagreement, and disagreement. In each group, the confidence of AI was expressed as ratio of the three AI confidence: confident, moderately confident, or unconfident. This AI confidence can be compared to the self-confidence of AI and that of humans in the classifying process.

### Statistical analyses

All statistical analyses were performed with SciPy on python (version 0.16.1, https://www.scipy.org/scipylib/). Comparisons of the age, visual acuity, refractive error (spherical equivalent), axial length, central choroidal thickness (CCT), and performance evaluations between AI models were performed by Mann-Whitney U test. A *P* value <0.05 was taken to be significant.

## Results

### Differences in background between groups

Of the 451 eyes, 413 eyes were classified as agreements by the two raters, and these eyes were subjected to the following analyses. Among them, 105 were pachychoroid spectrum disease patients, and 308 were healthy volunteers. In the 413 eyes, there were 204 symmetric and 209 asymmetric images (Tables 2 and 3). Mann–Whitney U tests showed that the age, visual acuity, refractive error, axial length, and central choroidal thickness (CCT) were significantly different between the symmetry and asymmetry image groups (all *P* <0.01; Table 3).

**Table 2:** Breakdown of symmetric and asymmetric patterns.

	Normal	Pachychoroid spectrum disease	Total
**Symmetry**	188	16	204
**Asymmetry**	120	89	209

**Table 3:** Demographic data of studied eyes.

	Symmetry	Asymmetry	*P*-value
Age	38.3±17.3	48.8±18.1	<0.001
Visual acuity (logMAR)	-0.07±0.12	-0.03±0.17	<0.001
Refractive Error (Spherical equivalent) (D)	-2.70±2.65	-1.23±2.65	<0.001
Axial length (mm)	24.7±1.5	23.8±1.5	<0.001
Central choroidal thickness (μm)	262±79	348±105	<0.001

### Evaluation by each AI-powered model

**Fig 5:**
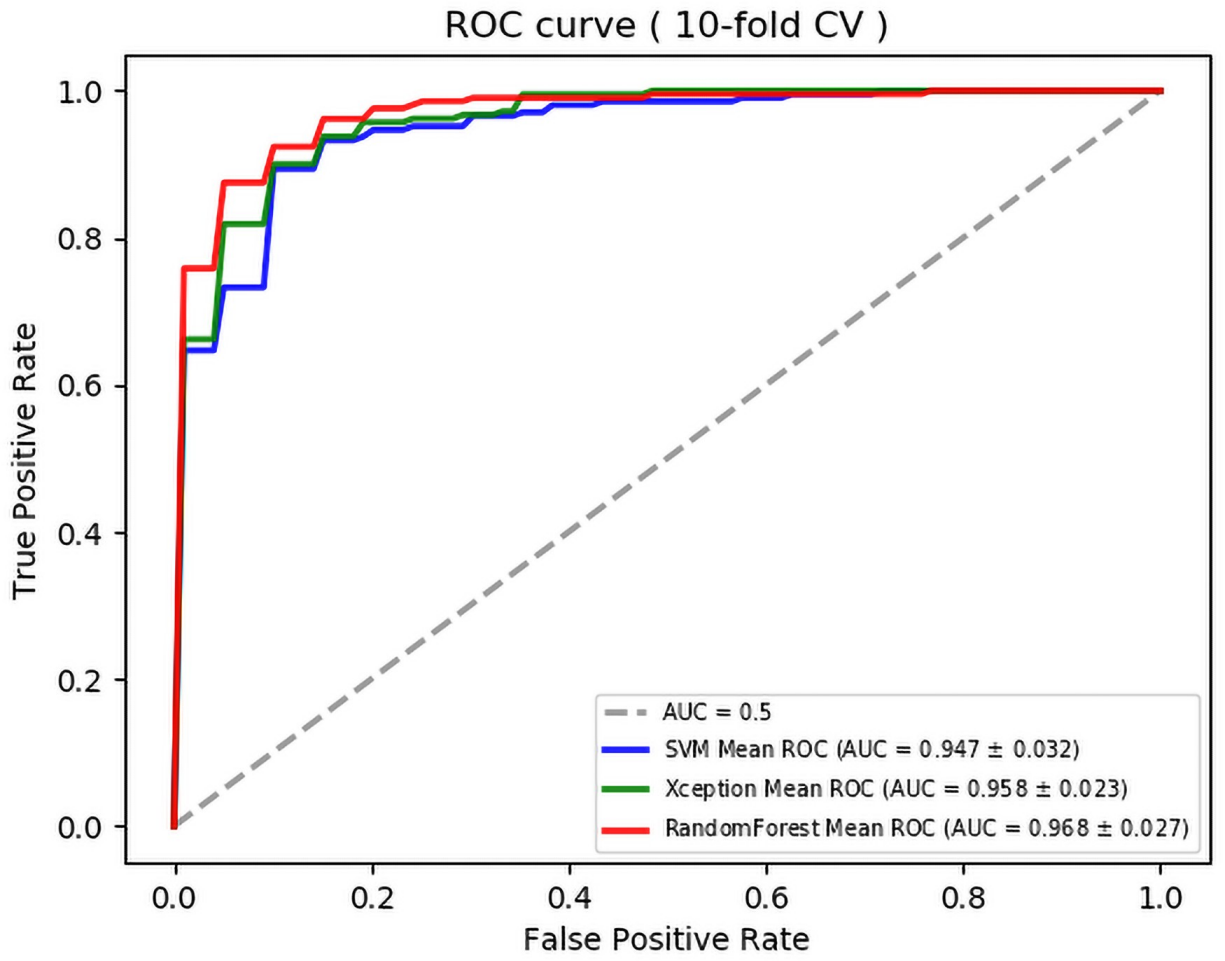
Mean receiver operationg characteristic curve (ROC) of 10-fold cross validations. The dotted line represents the trade-off resulting from random chance. The blue, green, and red curves represent the SVM, Xception, and Random Forest Models respectively. AUC = area under the receiver operating characteristic curve.

The 10-fold cross validation results for each of the three model are summarized in Table 4. We set asymmetry as positive and symmetry as negative, and we set the threshold to 0.5. All three models can classify the running pattern as symmetrical and asymmetrical with a high AUC (Fig 5).

**Table 4:** Results of 10-fold cross validation for each of the three models.

	AUC	Accuracy	Recall	Precision
**SVM**	0.947±0.032	0.906±0.043	0.899±0.063	0.914±0.050
**Xception**	0.958±0.023	0.889±0.061	0.891±0.084	0.891±0.066
**Random Forest**	0.968±0.027	0.911±0.047	0.919±0.044	0.908±0.063

**Fig 6:**
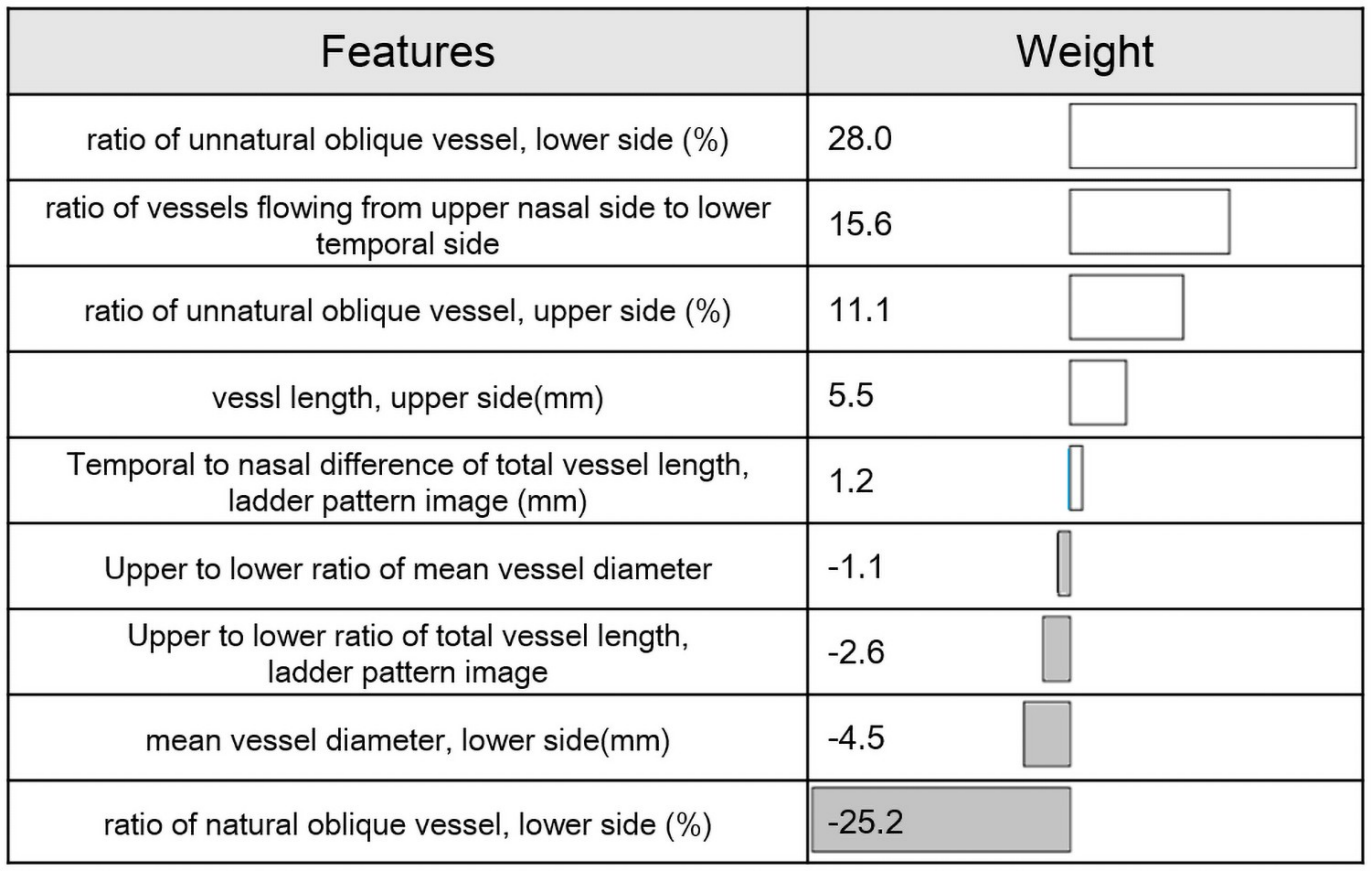
Selected features and contribution to the discrimination. The weights of the top 9 features in the process of SVM model obtaining answers.

The SVM using GAFS had a higher classification performance with an accuracy of 90.6% and kappa of 0.812 than without using GAFS which had an accuracy of 87.4% and kappa of 0.748. The 9 most effective features were selected, and the contribution of these features to identify the asymmetry of the en face images were quantified with the trained SVM (Fig 6).

The top 10 contributors to the 128 feature quantities of the Random Forest with the highest AUC are shown in Table 5. These values are the means of a 10-fold cross validation process. The largest contributor was the 1st principal component created from the feature quantities extracted from the Xception model which was more than five times greater than that of the second-place feature.

**Table 5:** Top 10 contributions of feature quantities to the Random Forest.

Features	Weight
**1st principal component from Xception**	0.282±0.006
**Ratio of natural to unnatural oblique vessel, upper side**	0.053±0.004
**mean vessel diameter, lower side**	0.035±0.002
**Ratio of natural to unnatural oblique vessel, lower side**	0.030±0.002
**mean vessel diameter, upper side**	0.026±0.002
**Upper to lower difference of mean vessel diameter**	0.023±0.003
**ratio of natural oblique vessel, upper side**	0.022±0.002
**3rd principal component from Xception**	0.021±0.022
**ratio of unnatural oblique vessel, upper side**	0.021±0.003
**Upper to lower ratio of mean vessel diameter**	0.018±0.003

### Comparisons of three methods

The Wilcoxon signed-rank test was used to determine the AUC and accuracy, and we assumed that there was a significant difference when the *P* value was <0.05. The results showed that there were no significant differences in the AUC and accuracy between the results of the SVM and Xception models.

Next, we compared the Random Forest, SVM, and Xception models. The AUC and accuracy of the Random Forest model were significantly higher than those of the SVM and Xception models. In addition, the *P*-value for AUC between SVM and random forest was 0.0080 and that of accuracy between Xception and Random Forest was 0.0269. Therefore, the Random Forest model was significantly more accurate than the SVM model in terms of the AUC and Xception (Tables 4 and 6).

**Table 6:** Performance comparison between AI models *(P-*value of Wilcoxon signed-rank test).

	AUC	Accuracy
**SVM/Xception**	0.285	0.398
**SVM/RandomForest**	0.00800	0.575
**Xception/RandomForest**	0.114	0.0269

We achieved high AUC and accuracy with the SVM model by using the feature quantities constructed by the retina specialists. We also achieved the AUC and accuracy as high as that of SVM by the Xception model. The input of the Xception was only the en-face images which means that without relying on doctors, important feature quantities could be created for the classification of the images into symmetrical and asymmetrical groups. Furthermore, by combining the feature quantities constructed by doctors and those extracted from Xception, we created the Random Forest model which was significantly better than the SVM and Xception models.

### Concordance of classification between AI models and humans

**Fig 7:**
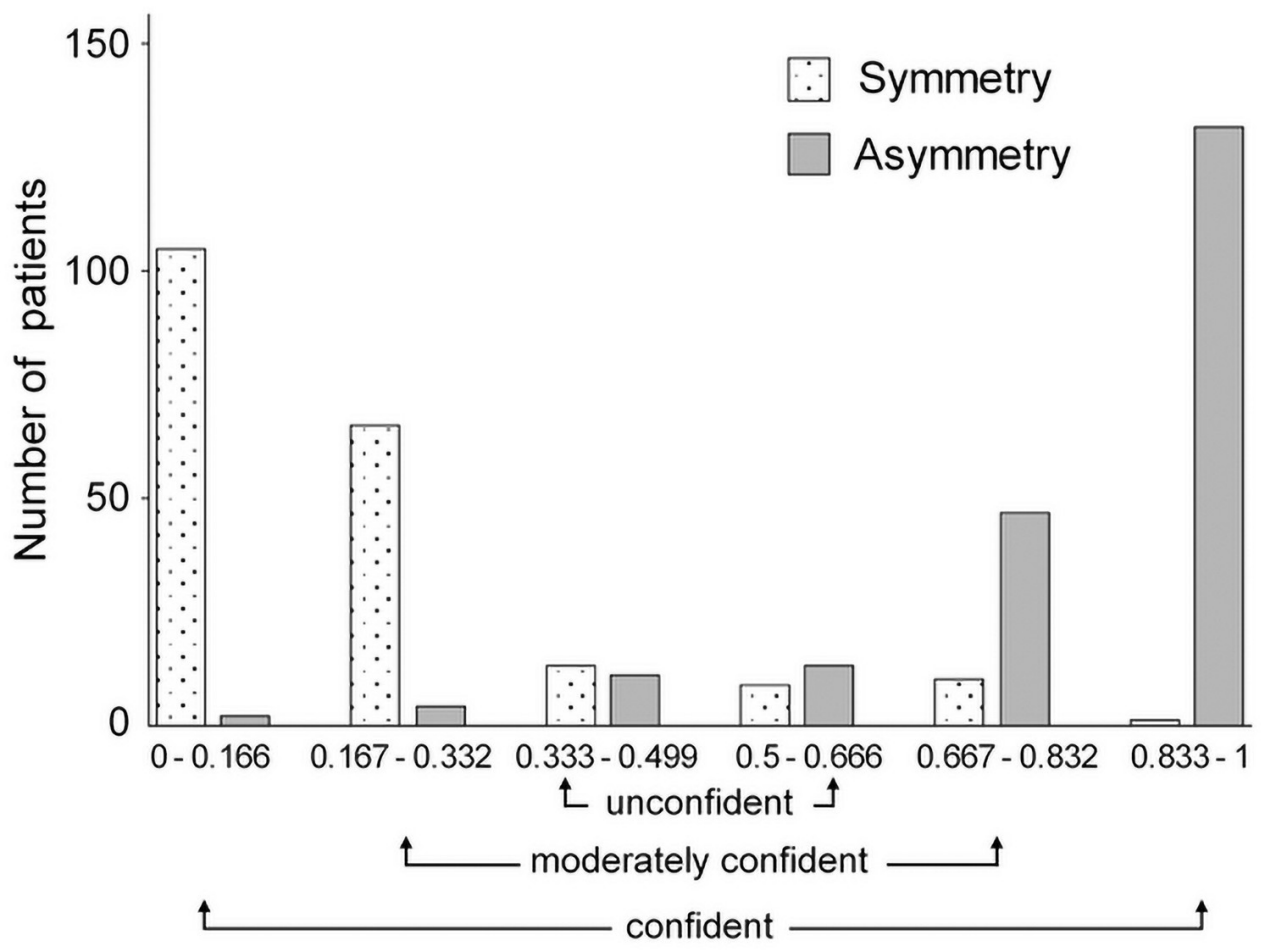
Confidence score (CS) for symmetric and asymmetric groups. The CS value was between 0 and 1, and the closer CS was to 0, the more confident it was asymmetrical, and the closer CS was to 1, the more confident it was to be symmetrical.

The unconfident group had 22 eyes in the symmetry group and 24 eyes in the asymmetry group. The moderate confidence group had 76 eyes in the symmetry group and 51 eyes in the asymmetry group. The confident category group had 106 eyes in the symmetry group and 134 eyes in the asymmetry group (Fig 7). The three graders classified 244 cases (53.8%) into the agreement group, 90 cases (21.8%) into the partial agreement group, 57 cases (13.8%) into the partial disagreement group, and 22 cases (5.3%) into the disagreement group.

**Fig 8:**
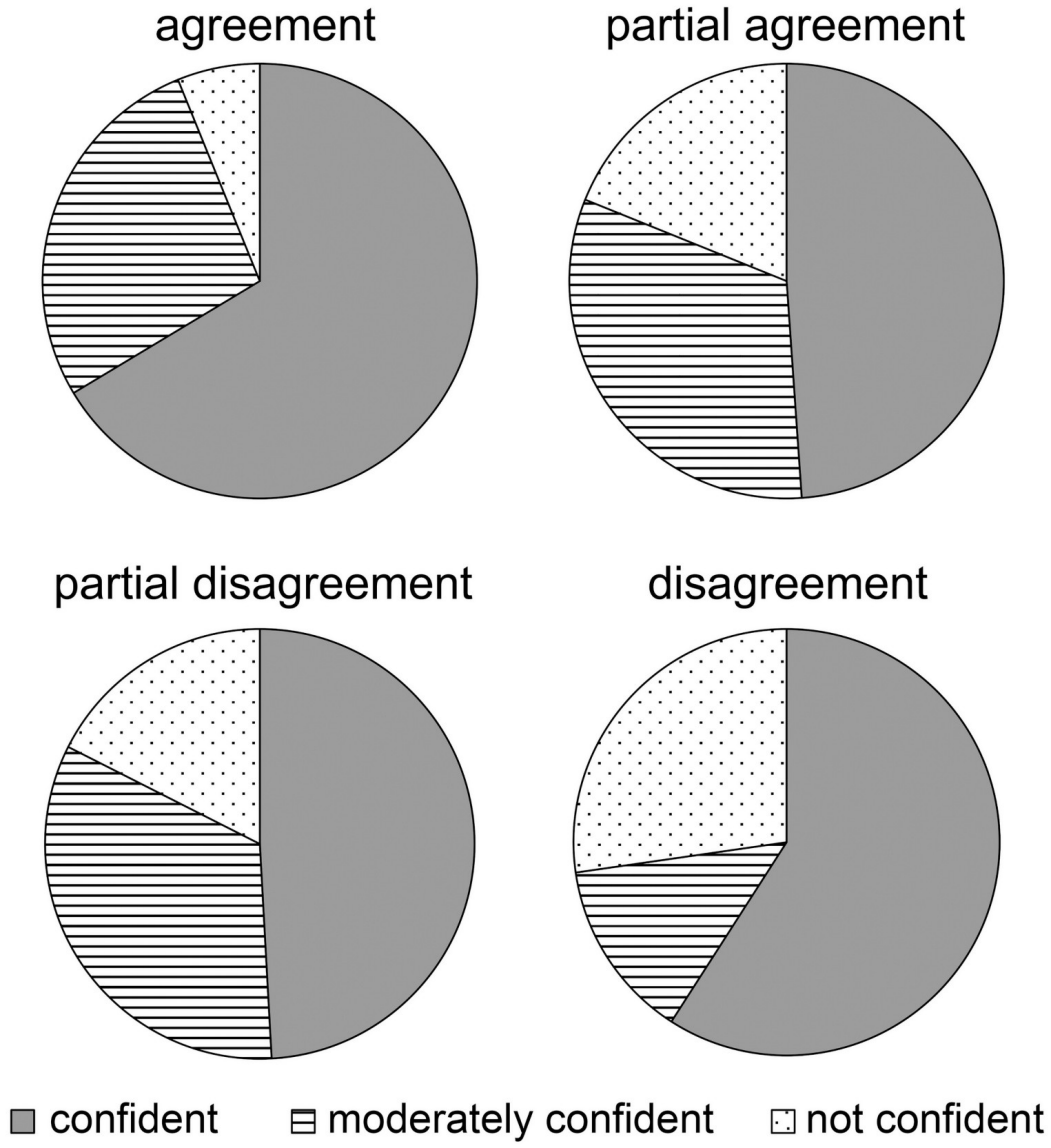
Breakdown of degree of certainty in the judgment of each group. In the agreement group in which all three examiners answered correctly, AI also had the highest percentage of confident and not confident. On the other hand, in the disagreement group in which all examiners had incorrect answers, AI indicated the highest proportion of not confident.

In the agreement group, the Xception model classified 6.1% into unconfident, 27.5% into moderately confident, and 66.4% into confident. In the partial agreement group, the model classified 18.9% into unconfident, 32.2% into moderately confident, and 48.9% into confident. In the partial disagreement group, the model classified 17.5% into unconfident, 33.3% into moderately confident, and 49.1% into confident. In the disagreement group, the model classified 27.3% into unconfident, 13.6% into moderately confident, and 59.1% into confident. Thus, the proportion of cases classified as unconfident was low in the agreement group and high in the disagreement group (Fig 8).

## Discussion

In spite of the widespread use of OCT in ophthalmological imaging, the analyses of choroidal en face images are not easy. First, there is an absence of fixed reference points such as the fovea and the optic disc as seen in OCT B-scan images. Second the choroid does not have a well-organized architecture as the retina [13]. Thus, it is difficult to classify morphological differences of an ambiguous structure into clusters [26]. Therefore, we compared the performance of AI and humans on determining the symmetry of the choroidal vascular running pattern. Additionally, to minimize the ambiguity of the supervised data, the supervised data were limited only to the ones in which the two experienced graders agreed on. Thus, the 38 cases (8.2%) that had inconsistent classification between the graders were excluded from the analyses.

To resolve this problem, we first quantified the numbers that can be quantified by humans and then classified them with the SVM model. Next, the Xception model, which does not require human quantification, was used to classify the problem. To improve the discrimination accuracy further, the features that were important in each model were combined to create a random forest model. All three AI-powered algorithms had high judgment accuracy. Each of the three models could discriminate the presence or absence of symmetry in the blood vessel running pattern with high accuracy with an AUC score of >0.94 (Table 4, Fig 5).

We found several advantages and disadvantages in each model. The supervised CNN model generally required a large number of cases, and a high AUC was attained with a smaller number of 413 cases with the Xception model [22] which is consistent with an earlier report [18]. Most importantly, unlike SVM, creating a CNN model required only the en face images of Xception which is a great advantage.

The average value of AUC of the Random Forest model was found to be higher than that of the SVM or Xception models. The Random Forest model performed only two tasks: calculation of the feature quantities of the images to lead to a correct answer and classification based on them [23, 27]. The combination of features extracted by Xception and the 28 uniquely designed features used in our SVM model were used for the Random Forest model. This then probably enhanced its performance. More interestingly, the feature quantities considered most important by the Random Forest model were not used by humans but by the Xception model (Table 5). In AI studies, it has been reported that a combination of appropriate feature values and models can provide higher accuracy [28, 29]. This means that the combination method was effective in the choroidal image analysis using AI. The Random Forest model had the highest discrimination performance, but all models had sufficient capabilities with high AUC values. It has been reported that the accuracy of CNN models can be improved by combining them [30–32]. Although only one CNN model was used in this study, it may be possible to improve the accuracy further by combining multiple CNN models.

Each model has advantages and disadvantages which should be considered in selecting the optimal AI model for each purpose. Of importance was that the characteristics of the AI was compared to that obtained by three examiners who were not the graders of the supervised data of the 413 cases. The results were compared with the supervised data which were used as the ground truth. The results showed that the rate of correct answers of the AI was higher than that of any of the three examiners. Thus, it is possible for AI to make a correct assessment more accurately than humans. This is consistent with an earlier report for AI to grade conjunctival hyperemia [8]. This suggests that AI is better in classifying ambiguous items than humans.

Of note, comparisons of the CSs were carried out [24, 33]. CS is an index which quantifies the degree of confidence in answers given by AI. In a case with which the three human examiners agreed, the CS was high. In a case in which one or more examiners made a mistaken judgment, the CS was low. Thus, if the supervised data and the independent evaluator’s opinion agree, the AI will make a classification with confidence, but if the supervised data and the independent evaluator’s judgment do not match, the AI will also have no confidence in the classification. This suggests that the thinking processes of AI is similar to that of humans. On the other hand, compared with the partial agreement and partial disagreement groups where judgments were split among independent evaluators, the AI of the disagreement group in which all the independent evaluator disagreed with the supervised data gave the answer with confidence as well. It is possible that AI follows a thinking process different from that of humans. In operating AI that assists in human decision making, the index of confidence of AI will be helpful to determine how much we should adopt AI in critical decisions.

In this study, only one eye was analyzed in each subject. In the patients with diseased eyes, fundus images were usually taken from both eyes in practice. However, fundus images were not always taken from both eyes in the normal cases due to the ethical reason. In addition, it is reported that using both eyes in the analysis may lead to statistical errors*. Thus, we decided to use one eye in all cases.

It is generally assumed that the choroidal vascular structure is an essential factor in the pathogenesis of the pachychoroid spectrum disease. However, because of the complicated structure of the choroid, previous studies subjectively analyzed choroidal structure. On the other hand, AI models could automatically classify the ambiguous types of choroidal vessel running patterns with high repeatability [15]. Because diagnosis or treatment for the retinochoroidal diseases such as AMD and CSC differs depending upon choroidal structure [34, 35], our AI model might be helpful for clinicians to judge whether patients have choroidal abnormality or not.

There are limitations in this study. The study population consisted of healthy eyes and diseased eyes and each had different backgrounds such as age, refractive power, and axial length. The digitalization of the vector components related to choroidal vessel running pattern using our proprietary software revealed that factors such as age and refractive power do not have a significant effect. Therefore, we do not consider the effect of backgrounds to be significant. In addition, there were two evaluators for the supervised data, and the study was performed at a single institution. To adopt this method and generalize the present results, verifications at other institutions are required. Thus, it is necessary to verify whether our AI model can exhibit the same high accuracy with data from other facilities.

## Conclusions

It was possible to develop an AI algorithm that automatically determines the presence or absence of symmetry of the blood vessel running pattern in the choroidal en face images. The use of AI in cases with subjective or ambiguous classifications will enable more objective evaluations, and it is expected to promote better objective classifications.

## Supporting information

S1 Fig Representative cases. The en-face image of Haller’s layer of the right eye of a 40-year-old man with no apparent pathologic findings. The vessel running pattern is symmetrical with respect to the macula at the center and shows a high proportion of natural oblique vessel in both areas (upper row). A 38-year-old man with central serous chorioretinopathy. The vessel running pattern at the posterior pole of the fundus is asymmetrical, and the proportion of the unnatural oblique vessel is high in the upper area (lower row). The natural oblique vessels are shown in blue and the unnatural oblique vessels are shown in red.
(TIF)

S1 Dataset Minimal dataset. (XLSX)
