# Supplementary figures and images for: Artificial intelligence for classifying uncertain images by humans in determining choroidal vascular running pattern and comparisons with automated classification between artificial intelligence
Source: PLoS One. 2021 May 14;16(5):e0251553. doi: 10.1371/journal.pone.0251553 (PMC8121314; doi:10.1371/journal.pone.0251553)

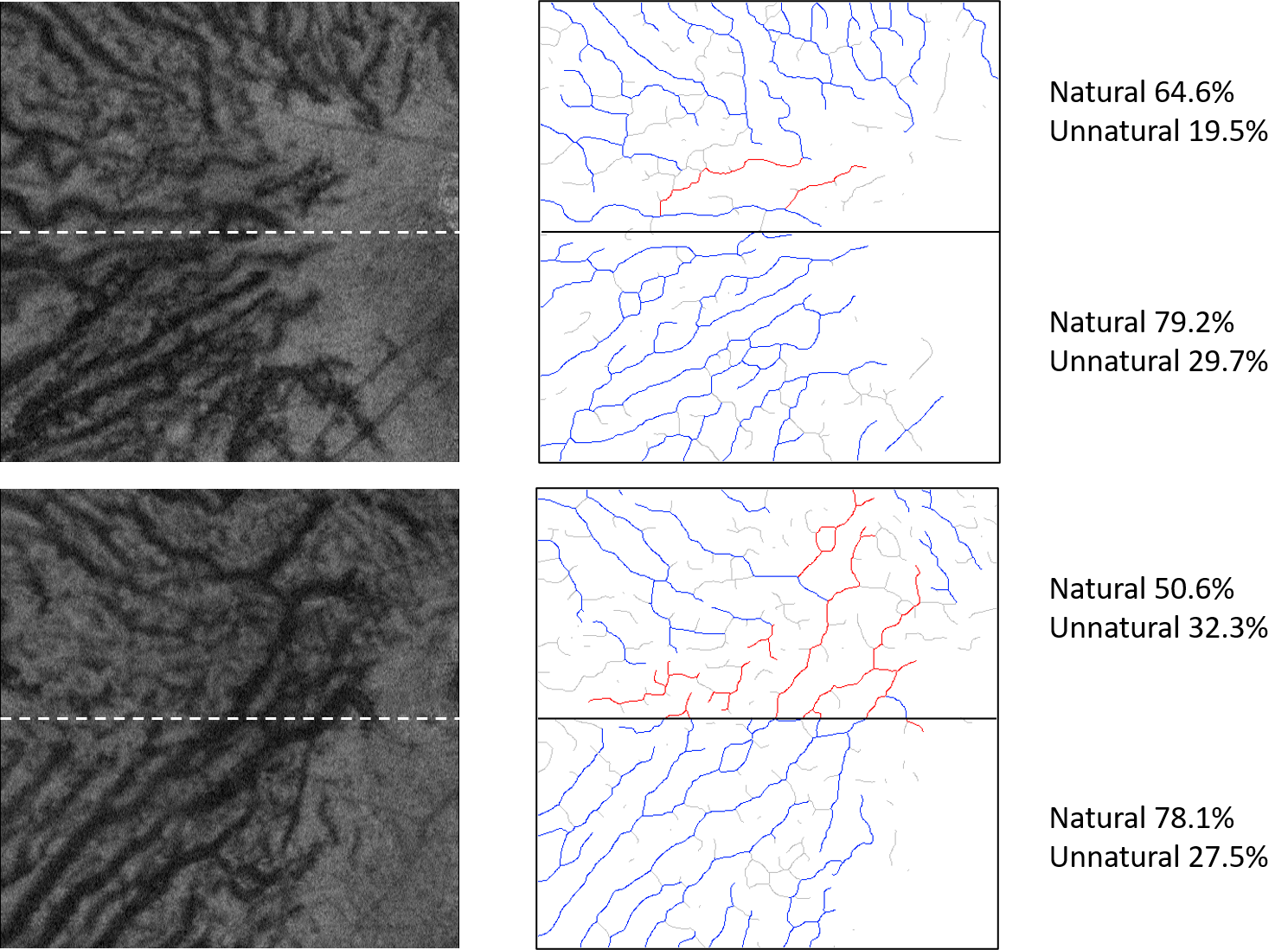

Supplement: S1 Fig — The en-face image of Haller’s layer of the right eye of a 40-year-old man with no apparent pathologic findings. The vessel running pattern is symmetrical with respect to the macula at the center and shows a high proportion of natural oblique vessel in both areas (upper row). A 38-year-old man with central serous chorioretinopathy. The vessel running pattern at the posterior pole of the fundus is asymmetrical, and the proportion of the unnatural oblique vessel is high in the upper area (lower row). The natural oblique vessels are shown in blue and the unnatural oblique vessels are shown in red. (TIF) [file pone.0251553.s001.tif]
